# Enhanced Activity of Supported Ni Catalysts Promoted by Pt for Rapid Reduction of Aromatic Nitro Compounds

**DOI:** 10.3390/nano6060103

**Published:** 2016-06-04

**Authors:** Huishan Shang, Kecheng Pan, Lu Zhang, Bing Zhang, Xu Xiang

**Affiliations:** 1 State Key Laboratory of Chemical Resource Engineering, Beijing University of Chemical Technology, Beijing 100029, China; huishan6880220@163.com (H.S.); xiangxubit@sohu.com (K.P.); zhanglu@mail.buct.edu.cn (L.Z.); 2 School of Chemical Engineering, Zhengzhou University, Zhengzhou 450001, China

**Keywords:** supported catalysts, heterogeneous catalysis, nickel, platinum, reduction

## Abstract

To improve the activities of non-noble metal catalysts is highly desirable and valuable to the reduced use of noble metal resources. In this work, the supported nickel (Ni) and nickel-platinum (NiPt) nanocatalysts were derived from a layered double hydroxide/carbon composite precursor. The catalysts were characterized and the role of Pt was analysed using X-ray diffraction (XRD), high-resolution transmission electron microscopy (HRTEM), energy dispersive X-ray spectroscopy (EDS) mapping, and X-ray photoelectron spectroscopy (XPS) techniques. The Ni^2+^ was reduced to metallic Ni^0^ via a self-reduction way utilizing the carbon as a reducing agent. The average sizes of the Ni particles in the NiPt catalysts were smaller than that in the supported Ni catalyst. The electronic structure of Ni was affected by the incorporation of Pt. The optimal NiPt catalysts exhibited remarkably improved activity toward the reduction of nitrophenol, which has an apparent rate constant (*K*_a_) of 18.82 × 10^−3^ s^−1^, 6.2 times larger than that of Ni catalyst and also larger than most of the reported values of noble-metal and bimetallic catalysts. The enhanced activity could be ascribed to the modification to the electronic structure of Ni by Pt and the effect of exposed crystal planes.

## 1. Introduction

Aromatic nitro compounds are widely generated as byproducts in various industries, including in the production of pigments, pesticides and medicines [1]. 4-nitrophenol (4-NP) is among the most common aromatic nitro compounds, and is harmful to the environment [2]. 4-aminophenol (4-AP), the reduction product of 4-NP, is an important intermediate for the manufacture of dyes, agrochemicals, and pharmaceuticals [3,4,5].

Various methods to synthesize 4-AP have been reported, such as multi-step iron-acid reduction of 4-NP, catalytic reduction of nitrobenzene, and electrochemical synthesis [6,7,8]. Among these methods, catalytic reduction is an alternative green process for 4-AP production because it does not generate a large amount of un-reusable Fe–FeO sludges and acid/alkali effluents [9]. It is well known that diverse noble metal such as palladium, platinum, and gold catalysts have been widely used in the catalytic reactions due to their high catalytic activities [10,11,12,13]. However, the high cost and scarcity in nature limit their practical applications. Therefore, non-noble metal catalysts have been paid more attention because of their abundance and reduced cost [14]. As we know, the reduction rates of 4-NP to 4-AP over non-noble metal catalysts are far slower than noble metal ones [15]. Therefore, it is desirable to improve the catalytic activities of the supported non-noble metal catalysts.

Among non-noble metals, nickel nanoparticles (Ni NPs) have drawn much attention because of their easy availability, relatively high catalytic activity, and magnetic separation feature [16,17]. Moreover, the unique physical and chemical properties can be introduced by adding a small amount of second elements such as Pd, Pt, Ir, or Ru to Ni catalysts. These elements are dispersed on the surface of Ni to form bimetallic catalysts and the interactions at the metal–metal interface lead to the improved performance of Ni catalysts [18,19,20,21]. Thus, a promising endeavor is to explore a combination of noble metal, e.g., Pt and Ni NPs to enhance the catalytic activity. In addition, the Ni-based catalysts can be easily separated from the reaction medium by application of an external magnetic field.

In the previous work, we prepared supported Ni catalysts via self-reduction of hybrid NiAl-layered double hydroxide/carbon (NiAl-LDH/C) composites [22]. The composites were assembled via crystallization of LDH in combination with simultaneous carbonization of glucose under hydrothermal conditions. The resulting carbon acted as a reducing agent to convert nickel oxide to metallic nickel upon calcination. The as-synthesized Ni nanoparticles had small crystallite sizes and high dispersions in the support owing to the confined effects of LDH and carbon. Inspired by this finding, we extended this method to prepare Pt-modified Ni catalysts. The glucose-derived carbon in the composites led to *in situ* reduction of chloroplatinic acid anions upon hydrothermal carbonization and thus introduced Pt to the Ni catalysts. The catalytic activities of the resultant Pt-modified Ni catalysts for liquid-phase reduction of 4-NP to 4-AP were investigated under mild reaction conditions. The optimal NiPt catalysts exhibited larger apparent rate constant (*K*_a_) than most of the reported values of noble-metal and bimetallic catalysts.

## 2. Results and Discussions

### 2.1. Characterization of Materials

**Figure 1:**
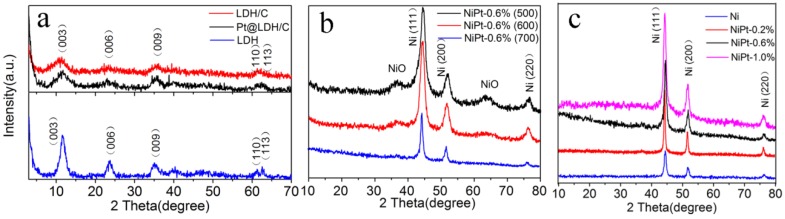
X-ray diffraction (XRD) patterns of (**a**) layered double hydroxide (LDH), LDH/carbon (C), and Pt@LDH/C; (**b**) NiPt catalysts obtained at different calcination temperatures; and (**c**) the Ni and NiPt catalysts with different Pt loadings.

The XRD patterns of LDH, hybrid LDH/C and Pt@LDH/C composites were shown in Figure 1a. The pattern of LDH sample exhibited the characteristic (003), (006), (009), (110), and (113) reflections, corresponding to layered hydrotalcite-like compounds [23]. The intensive reflections revealed the highly crystalline nature of the product. In contrast, the patterns of LDH/C and Pt@LDH/C composites presented broadened reflections at the same 2θ positions as those of LDH. It was noted that no graphite or other forms of carbon phases were detected, suggesting that carbon products mostly existed in amorphous form. The broader and weaker reflections for LDH/C and Pt@LDH/C were caused by both the dilution effect of the resultant carbon component in the composites and the reduced crystalline feature of LDH phase. No reflections associated with metal Pt or other Pt species were observed in Pt@LDH/C. This finding could be ascribed to the high dispersion of the Pt nanoparticles of small sizes and/or the very low loading [24]. It was believed that the aromatization and carbonization of glucose yielded amorphous carbon under the present hydrothermal treatment [25], and the resultant carbon was assembled with LDH crystallites during the crystallization of LDH, thereby leading to the formation of hybrid composites.

The effects of calcination temperatures on the phase structure were also studied. Figure 1b showed XRD patterns of Pt@LDH/C precursor calcined at 500 °C, 600 °C, and 700 °C in a flowing N_2_ atmosphere. It was known that the layered structure of LDH collapses and is converted into mixed-oxide phases when heating above 450 °C [26]. For NiPt-0.6% (500) and NiPt-0.6% (600) samples, the XRD patterns exhibited the co-existence of Ni and NiO phases. Furthermore, the relative intensity from the reflections of NiO decreased with increasing calcination temperature. After elevating the temperature to 700 °C, only the contributions from metallic Ni were observed, which can be indexed to the face-centered cubic (fcc) Ni phase (JCPDS No. 87-0712). The three peaks centered at 2θ = 44.4°, 51.7° and 76.5° corresponded to the characteristic (111), (200) and (220) planes of Ni phase. The results indicated that the complete phase transformation from NiO to Ni occurred at this temperature.

The LDH-derived metal oxides could be reduced into metal via self-reduction by carbon in the LDH/C composite, leading to the tranformation of Ni^2+^ to Ni^0^. The main reactions can be expressed as follows:

(1) NiO + C (s)→ Ni + CO (g)

(2) $$\text{NiO }+\text{ CO }\left(g\right)\to \text{Ni }+{\text{ CO}}_{2}\;\left(g\right)$$

During heating, LDH was transformed into mixed oxides (crystalline NiO and amorphous alumina). After reduction, the residual carbon acted not only as the support but also as the dispersing matrix, which prevented Ni particles from agglomeration. Figure 1c showed the XRD patterns of NiPt catalysts with different Pt loadings. These XRD patterns of the Ni and NiPt samples were hardly distinguishable, which could indicate that the Pt species were highly dispersed in the NiPt catalysts [27].

**Figure 2:**
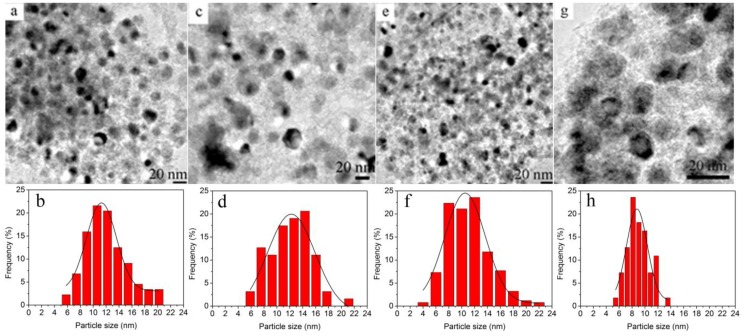
Transmission electron microscopy (TEM) images and size distributions of the supported Ni and NiPt catalysts with different Pt loadings (**a**) and (**b**) Ni; (**c**) and (**d**) NiPt-0.2%; (**e**) and (**f**) NiPt-0.6%; (**g**) and (**h**) NiPt-1.0%.

TEM observations were carried out to investigate the morphologies and microstructures of NiPt catalysts with different Pt loadings (Figure 2). TEM-derived histograms of the Ni particle size distributions were presented. The TEM images indicated that the Ni nanoparticles were well dispersed on the support and the size slightly decreased from 12.1 to 11.0 nm when the nominal Pt content was increased to 0.6% compared to the pristine Ni. When the Pt content was increased to 1.0%, the Ni nanoparticles had smaller size (~9 nm) and narrower size distributions (Table 1). The Ni loading reached as high as 36%–39% whereas no obvious aggregates were observed, suggestive of excellent metal dispersion. The Pt particles could not be found in the TEM images owing to the low loading. The Pt loading measured was lower than the nominal value, possibly due to the metal leaching during the hydrothermal reactions (Table 1). One can find that these catalysts had similar specific surface areas, which indicated that the surface area could not be the dominant factor to adjust the catalytic properties [28].

**Figure 3:**
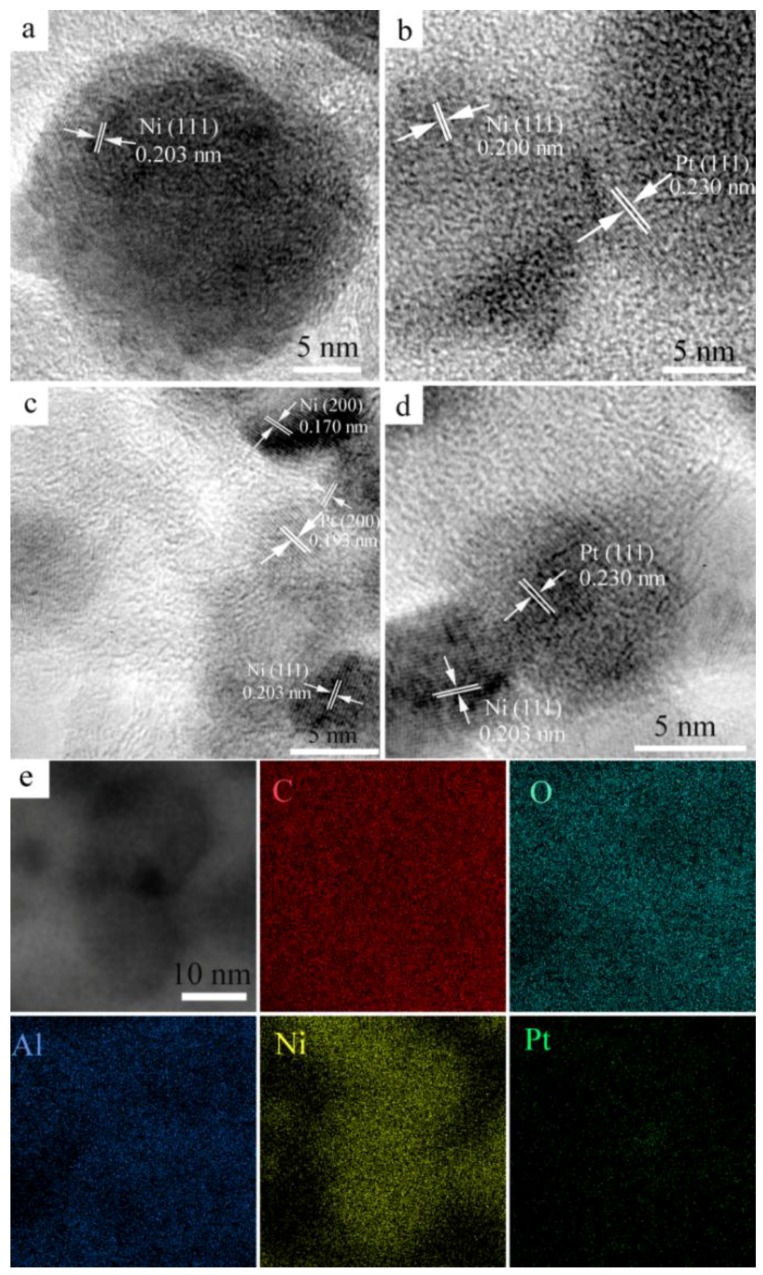
High-resolution transmission electron microscopy (HRTEM) images of the supported catalysts: (**a**) Ni; (**b**) NiPt-0.2%; (**c**) NiPt-0.6%; (**d**) NiPt-1.0%; (**e**) Energy dispersive X-ray spectroscopy (EDS) mapping of the catalyst NiPt-0.6%.

To further reveal the microstructures of the Ni and Pt NPs, high-resolution transmission electron microscopy (HRTEM) observations were conducted. As shown in Figure 3a, the lattice spacing of the supported Ni NPs was measured to be 0.203 nm, consistent with the d-value of plane (111) of cubic phase Ni. Regarding NiPt-0.2% and NiPt-1.0% catalysts, the Ni NPs had the same d-spacing of 0.203 nm (Figure 3b,d), and the Pt NPs exhibited a d-spacing of 0.230 nm, corresponding to plane (111) of Pt. The adjacent interface could be observed between plane Ni (111) and Pt (111). As to NiPt-0.6% catalysts, the d-spacing of 0.170 nm for Ni NPs and 0.193 nm for Pt NPs were observed, which corresponded to Ni (200) and Pt (200), respectively (Figure 3c). It was interesting that the interface formed between Ni (111) and Pt (111) or Ni (200) and Pt (200). Such interface of different planes of Ni and Pt NPs could affect the catalytic properties. It is known that the catalytic reduction usually depends on the sizes, shapes, and/or exposed planes of the active metal NPs [23,29]. The differences in exposed planes and interfaces of NiPt catalysts could lead to distinct activities towards the reduction reaction. Energy dispersive X-ray spectroscopy (EDS) mapping analyses of NiPt-0.6% exhibited C, O, Al, Ni, and Pt elements (Figure 3e). It was seen that C, O and Al were homogeneously distributed in the whole matrix. The Ni components were highly dispersed on the matrix and as well the small amount of Pt.

**Figure 4:**
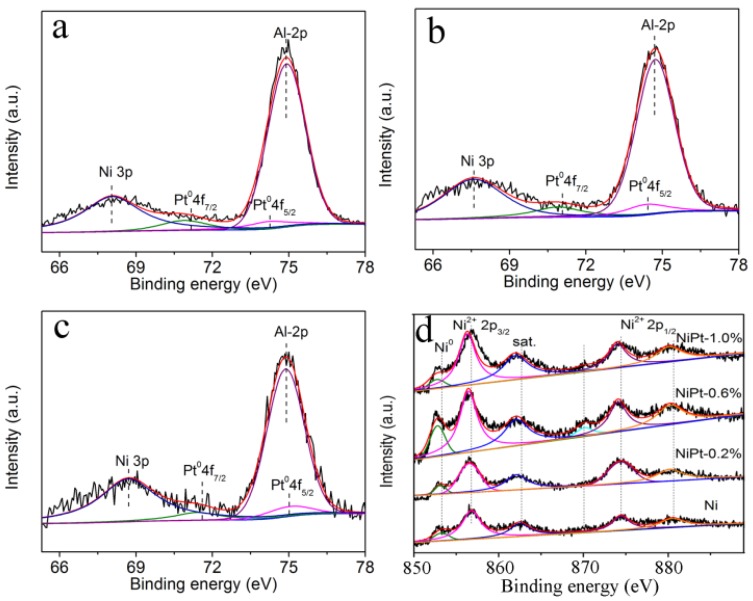
X-ray photoelectron spectroscopy (XPS) spectra of Pt and Ni core levels in the supported catalysts: (**a**) NiPt-0.2%; (**b**) NiPt-0.6%; (**c**) NiPt-1.0% and (**d**) Ni.

X-ray photoelectron spectroscopy (XPS) measurements were used to analyze the chemical states of the metal species in the catalysts. The XPS spectra of Pt and Ni core levels were shown in Figure 4. The Pt4f spectra could be deconvoluted into two peaks due to the spin-orbit splitting. One was at 70.9–71.4 eV, and the other was at 74.2–75.0 eV, which could be assigned to metallic Pt^0^ species [30,31]. As to NiPt-1.0% sample, the Pt4f peaks showed a 0.5–0.8 eV shift towards a higher binding energy compared to the other two NiPt catalysts (Table 2). This was an indication that the electronic structures of Pt were affected by the surrounding Ni NPs owing to the strong interactions between them [32]. The Ni2p spectra were deconvoluted into three contributions at ~853.3, ~856.8, and ~862.5 eV in the Ni2p_3/2_ region, which were assigned to the metallic Ni^0^, the Ni^2+^ species in NiO and the satellite, respectively [33]. The existence of Ni^2+^ species could be due to the easy oxidation of metallic Ni, which inevitably contact with the air during the sample preparation and settlement [22]. Compared to the Ni catalysts, the binding energy of Ni^0^ and Ni^2+^ (Ni2p_3/2_) in the NiPt-0.2% had a little shift of 0.1–0.2 eV towards a lower value. The binding energy shifted ~0.4 eV with the increasing Pt content in the NiPt-0.6% and NiPt-1.0% samples (Table 2). The binding energy of metallic Ni shifted to a lower value after a small amount of Pt was incorporated. It is a hint that the electronic structures of Ni were affected by Pt, suggesting the strong interactions between the two metal components. The binding energy at 68.9 eV could be the contribution from Ni3p [34,35]. The Ni3p peak overlapped with the Pt4f_7/2_ peak, which provided further evidence for the existence of interactions between Ni and Pt. The binding energy at 74.8 ± 0.1 eV was assigned to Al2p core level, which came from the Al_2_O_3_ matrix in the catalysts [36]. XPS characterization verified that the interactions between Ni and Pt were enhanced with the increasing content of Pt.

### 2.2. Evaluation of the Catalytic Activity

**Figure 5:**
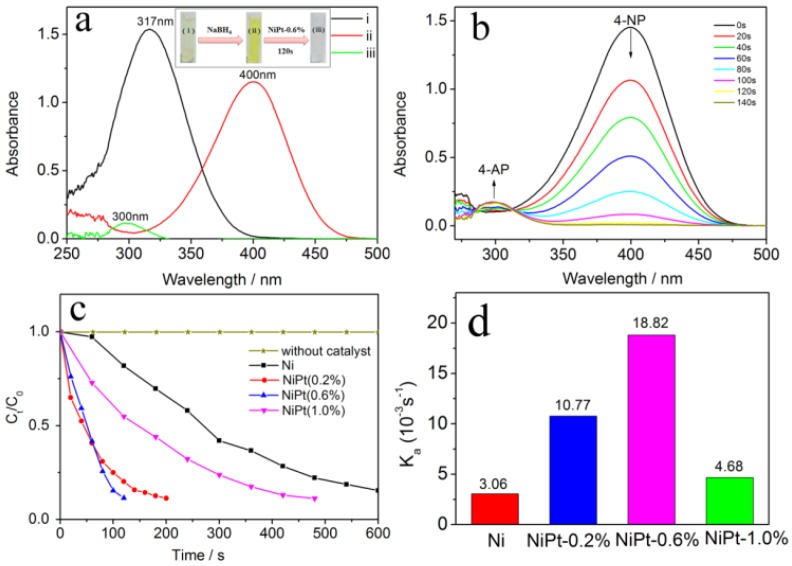
(**a**) Ultraviolet-visible (UV-VIS) absorption spectra of the solution before (i) and after (ii) the addition of NaBH_4_ and (iii) after the addition of NiPt-0.6% catalyst; (**b**) time-dependent UV-VIS absorption spectra of the reduction of 4-NP over the NiPt-0.6% catalyst in aqueous solution at room temperature; (**c**) reduction of 4-NP over different catalysts as a function of time; (**d**) apparent rate constants (*K_a_*) of the reactions in the presence of supported catalysts.

The catalytic activities of the as-prepared Ni and NiPt (0.2%, 0.6%, and 1.0%) catalysts were evaluated using a probe reaction, *i.e.*, the reduction of 4-NP into 4-AP. The reduction was conducted in an aqueous solution of 4-NP with the addition of NaBH_4_ as a reducing agent. The Ultraviolet-visible (UV-VIS) absorption spectra were recorded with time to monitor the transformation from 4-NP to 4-AP due to their distinct absorption positions. The original adsorption of 4-NP peaked at 317 nm (Figure 5a). The absorption shifted to 400 nm when the freshly prepared aqueous solution of NaBH_4_ was added, and the color of the solution immediately changed from the pristine light yellow to bright yellow (the inset of Figure 5a). This redshift was ascribed to the rapid formation of 4-nitrophenolate ions in alkaline solution upon the addition of NaBH_4_ [37]. When the NiPt-0.6% catalysts were added to the solution, the bright yellow solution faded and became colorless in tens of seconds. The absorption at 400 nm sharply decreased, and the absorption at 300 nm from 4-AP appeared [38]. The evolution of absorbance spectra with time clearly showed the conversion from 4-NP to 4-AP in the presence of NiPt-0.6% catalysts (Figure 5b). The reduction was completed within around 100 seconds according to the decreasing absorbance at 400 nm and the increasing absorbance at 300 nm, corresponding to the deletion of 4-NP and the formation of 4-AP, respectively.

The Ni and NiPt catalysts were compared based on the concentration changes of 4-NP as a function of time (Figure 5c). The reaction would not happen in the absence of catalysts. It was observed that the decrease in the concentration of 4-NP over the catalysts followed the order: NiPt-0.6% > NiPt-0.2% > NiPt-1.0% > Ni. The concentration of 4-NP decreased to 10% of the initial value within 120 seconds over NiPt-0.6% catalysts. It was noted that the optimal catalysts were NiPt-0.6% rather than NiPt-1.0% with a higher Pt content. This suggested that the activity of Ni catalysts could be enhanced by incorporating an appropriate amount of Pt. The excessive amount of Pt had a negative effect on the catalytic activity in the reduction of 4-NP to 4-AP. These findings were in agreement with the catalytic behaviors of AuCu alloy reported in a recent literature [39]. In that work, the reaction pathway for reduction of nitroaromatics to anilines was adjusted by tailoring the compositions of bimetallic nanocatalysts. A similar volcano curve associated with the yield of aniline was observed with the change of Cu contents. The volcano curve behavior indicated that the chemical compositions of the bimetallic nanocatalysts were important parameters for affecting the catalytic activity.

The reduction rate of 4-NP was independent of the concentration of reducing agent (NaBH_4_) since the initial concentration of NaBH_4_ greatly exceeded that of 4-NP (~100 times). Thus, the reduction process could be described with pseudo-first-order kinetics with respect to the concentration of 4-NP [40]:

(3) $$-\text{ln}\left(C_{t}/C_{0}\right)\;=\;K_{a}t$$

where *C*_0_ and *C_t_* (unit in mM) were the concentrations of 4-NP at the beginning and at a certain time, respectively. Figure 5d showed the apparent rate constants (*K*_a_) for different catalysts, which were calculated according to equation 3. The values of *K*_a_ for NiPt-0.2%, NiPt-0.6%, and NiPt-1.0% catalysts were 0.01077, 0.01882, and 0.00468 s^−1^, respectively, all of which were higher than that of the Ni catalysts (0.00306 s^−1^). This could be caused by the strong interactions between Ni and Pt. The electronic structures of Ni were modified by incorporation of Pt, which was consistent with the recent report on Ir-promoted Ni catalysts for hydrogenation reaction [18]. As to the different activities of NiPt catalysts, it might be due to the different exposed crystal planes of Ni NPs and Pt NPs. As shown in Figure 2c, the NiPt-0.6% catalysts showed the Ni (200) plane besides the Ni (111) one. The crystal plane-dependent activity has been confirmed in metal-supported oxide catalysts [41]. Further studies on the correlations were required by constructing a less complicated model catalyst.

In addition, the *K*_a_ values of various catalysts were compared in Table 3. It was seen that the NiPt-0.6% catalyst showed much larger *K*_a_ value than the reported nickel catalysts [4,6,14]. Importantly, this catalyst exhibited higher activity than noble-metal and bimetallic ones such as DPNs (dendritic Pt nanoparticles) [11], reduced graphene oxide (RGO)/PtNi [35], Pt/γ-Al_2_O_3_ [40] and Ni-Pt [42]. The comparisons further verified the superior catalytic activity of the optimal NiPt catalysts for 4-NP reduction and highlighted the very limited use of noble-metal Pt in the as-prepared high-performance catalysts.

Generally, the formation of a bimetallic structure can change the electronic states of metals, particularly the d-band center of the involved metal atoms, which is highly relevant to their catalytic activity [45]. The variations of the d-band in metals have been theoretically studied using density functional theory (DFT) computations assuming some specific extended-surface models [46,47]. Recently, the theory has also been applied to bimetallic systems to correlate the changes in the electronic states with the catalytic activities in a variety of reactions [48]. With regard to the NiPt NPs studied in this work, Ni was reduced *in situ* and enriched on the surface from a hybrid of LDH/carbon precursor. The pristine Ni NPs showed the activity towards the reduction of 4-NP. Upon introducing Pt to the Ni catalysts, the local electronic structures of the Ni were affected, and the electron density on the Ni was changed, which was dependent on the bimetallic compositions [42]. It was found that the NiPt-0.6% catalysts had higher activity than the NiPt-1.0% with a larger content of Pt. It could be due to the different exposed crystal planes of Ni NPs (Figure 2c).

**Figure 6:**
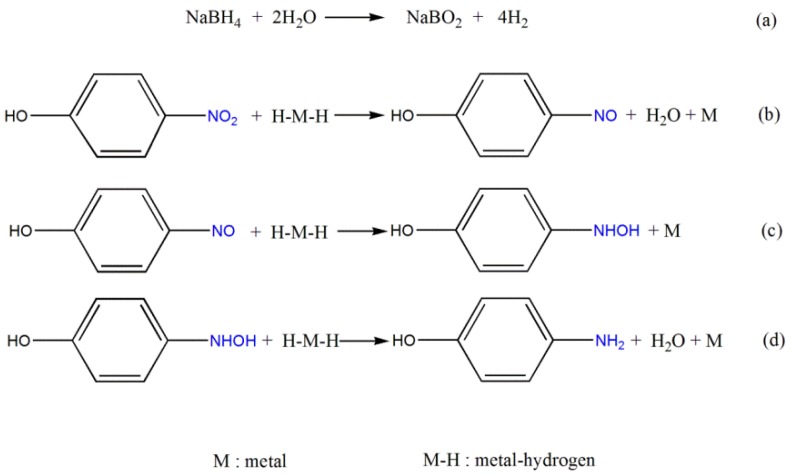
Reaction pathways for the reduction of 4-NP to 4-AP: (**a**) NaBH_4_ hydrolyzed to release H_2_; (**b**) H_2_ molecules split to H atoms on the surface of metal NPs and reacted with 4-NP; (**c**) H atoms reacted with the nitrosophenol intermediate to form hydroxylamine; (**d**) hydroxylamine was further reduced to the final product 4-AP. H–M–H represents the split of H_2_ molecules on the surface of metal NPs.

On the other hand, small nanoparticles and excellent dispersions resulted in high activity in many cases [49,50]. The sizes of nickel crystallites as estimated by TEM decreased with increasing amount of Pt. No obvious agglomeration of Ni NPs was observed although the Ni loading was very high (36%–39%). This highlighted the advantages of the precursor method from LDH/carbon hybrid owing to the lattice anchoring effect of metal ions in the layers of LDH [22,25]. The Pt-modified Ni NPs were more active for adsorbing and cleaving H_2_ molecules than the pristine Ni NPs during reduction of 4-NP, owing to a higher electron density of NiPt active sites [51]. The produced active hydrogen atoms reacted with 4-NP on the active sites to generate 4-AP. A reaction pathway was shown in Figure 6. First, NaBH_4_ rapidly reacted with water to release H_2_, in which the sodium metaborate (NaBO_2_) was formed as a byproduct. Then, the H_2_ split H–H bond into H atoms, which adsorbed on the surface of metal nanoparticles. There was no doubt that the increase in chemisorbed H atoms on NiPt nanoparticles could improve the reducibility. It was accepted that the more hydrogen adsorbed, the higher the catalytic activity exhibited [18]. The negatively-charged hydrogen in the metal–hydrogen bond attacked the positively charged nitrogen within the nitro group of 4-NP. Subsequently the nitro group was reduced to the nitroso group intermediate, followed by the reaction of two hydrogen atoms to form hydroxylamine. Finally, the hydroxylamine was further reduced to amino group to obtain 4-AP.

**Figure 7:**
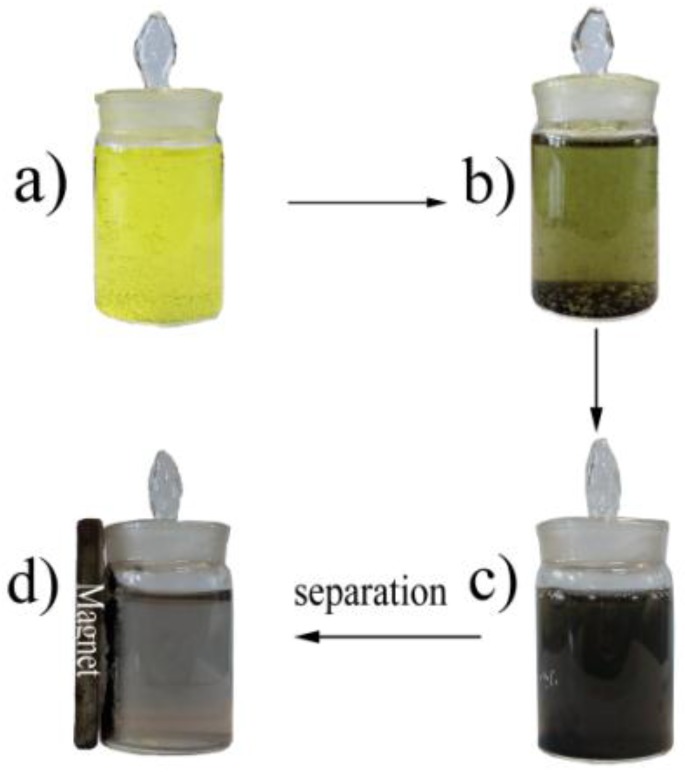
Separation of NiPt-0.6% from solution by a magnet: (**a**) before addition of the catalyst; (**b**) after addition of the catalyst; (**c**) the suspension solution after reaction; (**d**) separation of solid catalysts with a magnet.

The native magnetic property of the nanocatalysts caused by Ni made them economically and easily separated after reactions using an external magnet (Figure 7), and the catalysts were conveniently recycled and reused in a fresh solution.

## 3. Experimental Section

### 3.1. Materials

Hexachloroplatinic acid (H_2_PtCl_6_·6H_2_O, 99.9%) was purchased from Sinopharm Chemical Reagent Co., Ltd. (Shanghai, China). Glucose anhydrous and NaBH_4_ were purchased from Kermel Chemical Reagent Co., Ltd. (Tianjin, China). 4-nitrophenol, received from Macklin Biochemical Co., Ltd. (Shanghai, China), was used without further purification. Other chemicals and reagents were all analytical reagent grade and used as received without further purification. Deionized water was used throughout the process.

### 3.2. Synthesis of NiAl-LDH Precursor

A mixture of Ni(NO_3_)_2_·6H_2_O and Al(NO_3_)_3_·9H_2_O were dissolved in 30 mL of deionized water to form a clear salt solution ([Ni^2+^] = 0.2 M, [Al^3+^] = 0.1 M). The salt solution was rapidly poured into a 30 mL NaOH and Na_2_CO_3_ solution ([OH^−^] = 1.6[Ni^2+^ + Al^3+^], [CO_3_^2−^] = 2[Al^3+^]) within several tens of seconds under vigorous stirring. The mixture solution was further stirred for 10 min at room temperature. The resulting suspension was then centrifuged and re-dispersed in deionized water for five cycles to obtain LDH precipitate. The resulting precipitate was transferred and dispersed into a Teflon-lined autoclave (100 mL total volume), and 80 mL of deionized water was added to it. The autoclave was then tightly sealed and maintained at 150 °C for 10 h. The resulting bluish green suspension was directly vacuum-freeze-dried overnight to collect the solid NiAl-LDH powder.

### 3.3. Synthesis of LDH/Carbon Composites and Supported NiPt Catalysts

A certain amount of H_2_PtCl_6_·6H_2_O was added to 30 mL of glucose (C_6_H_12_O_6_) solution ([C_6_H_12_O_6_] = 2.5[Ni^2+^ + Al^3+^]). The precipitate obtained via the above-mentioned room-temperature co-precipitation was dispersed in the mixture solution. The resultant solution was transferred into an autoclave with the same fill volume and maintained at the same temperature (150 °C) for the same period of time (10 h). Subsequently, the product was centrifuged and washed with deionized water and ethanol five times. Finally, the precipitate was freeze-dried overnight to obtain LDH/C composites and Pt/LDH-C. The resulting composites were loaded into an alumina boat and calcined in a tube furnace in a flowing N_2_ atmosphere. The furnace was heated to a preset temperature at a rate of 5 °C/min, and the temperature was maintained for 2 h. After the reaction, the furnace was naturally cooled to room temperature. The obtained black product was denoted as NiPt-*x*, where *x* is the theoretical loading percentage of Pt (wt %).

### 3.4. Characterizations

UV-VIS absorption spectra were recorded using a (TU-1810 spectrometer, Purkinje, Inc., Beijing, China. TEM and high-resolution TEM (HRTEM) images were obtained using a JEM-2100 microscope (JEOL, Tokyo, Japan) with an Oxford INCA detector (Oxford-instruments, Shanghai, China) operating at 200 kV. The compositions of the as-prepared catalysts were determined through inductively coupled plasma-atomic emission spectroscopy (ICP-AES) (ICPS-7500 Spectrometer, Shimadzu, Inc., Beijing, China) Powder XRD analyses were performed using a Bruker D8 Advanced diffractometer (Bruker, Beijing, China) with Cu Kα radiation, and the scanning angle 2θ ranged from 5° to 80°. XPS measurements were performed using a VG ESCALAB250 (Thermo Fisher Scientific, MA, USA) with Al K_α_ radiation (hν = 1486.6 eV). The specific surface areas were measured via N_2_-adsorption at 77 K using a static volumetric Quantachrome Autosorb-1C-VP Analyser (Quantachrome, Shanghai, China). The specific surface area was calculated using a Brunauer–Emmett–Teller (BET) method.

### 3.5. Catalytic Evaluation

Catalytic reduction reactions of 4-nitrophenols were conducted at room temperature (25 °C) in the presence of NiPt catalysts with varying Pt content. Typically, aqueous solutions of 4-NP (2 mM) and NaBH_4_ (0.25 M) were freshly prepared. NiPt catalyst in the amount of 15.5 mg was dispersed into 40 mL of 4-NP and NaBH_4_ mixture solutions under continuous stirring. To evaluate the reaction progress, approximately 1 mL of solution was taken out of the reaction mixture at specified time intervals and subsequently diluted 20 times with deionized water. This step was followed by recording of the UV-VIS spectra of the solution to examine the concentration of 4-NP by monitoring the adsorption peak at 400 nm.

## 4. Conclusions

The supported NiPt nanocatalysts were synthesized by an *in situ* reduction strategy from Pt@LDH/carbon composites precursor. The precursor method ensured the formation of nanosized (~10 nm) and aggregate-free Ni NPs. The incorporation of Pt to the pristine Ni catalysts led to the enhanced activity towards the reduction of 4-NP to 4-AP because the modification of Pt affected the electronic structures of Ni. The optimal NiPt catalysts with an appropriate amount of Pt showed excellent activity in the reduction of 4-NP. The apparent rate constant (*K*_a_) reached 18.82 × 10^−^^3^ s^−1^, which was 6.2 times higher than that of Ni catalysts and much higher than most of reported catalysts in the literaures. The present method for bimetallic catalysts could be extended to other compositions owing to the adjustable components in LDHs to improve the catalytic activity of the monometallic ones and simultaneously to minimize the utilization of noble metals.

## Figures and Tables

**Table 1:** Metal loadings, sizes and specific surface areas of the supported catalysts.

Catalyst	Pt wt % ^1^	Ni wt %	D (nm) ^2^	Specific Surface Area (m^2^ g^−1^) ^3^
Ni	0	35.85	12.1	244.6
NiPt-0.2%	0.167	38.75	12.1	271.8
NiPt-0.6%	0.347	36.98	11.0	266.0
NiPt-1.0%	0.537	39.55	9.0	267.4

^1^ Pt content measured by inductively coupled plasma-atomic emission spectroscopy (ICP-AES); ^2^ The mean size of Ni nanoparticles (NPs) based on TEM analysis; ^3^ Specific surface area calculated by a Brunauer-Emmett-Teller (BET) method.

**Table 2:** XPS binding energy of the Ni, Pt and Al core levels in the supported catalysts.

Catalysts	2p_3/2_ (eV)			2p_1/2_ (eV)			4f_7/2_ (eV)	4f_5/2_ (eV)	Ni3p	Al2p
	Ni^0^	Ni^2+^	Ni^Sat.^	Ni^0^	Ni^2+^	Ni^Sat.^	Pt^0^			
Ni	853.3	856.8	862.5	870.9	874.4	880.5	-	-	-	74.8
NiPt-0.2%	853.2	856.6	862.1	870.1	874.4	880.2	70.9	74.2	68.1	74.9
NiPt-0.6%	852.8	856.4	862.1	870.1	874.1	880.3	70.9	74.4	67.7	74.7
NiPt-1.0%	852.7	856.3	862.0	870.0	874.0	880.1	71.4	75.0	68.4	74.9

**Table 3:** Comparisons of the apparent rate constant (*K_a_*) for 4-NP reduction over various catalysts.

Catalysts	Reaction Conditions ^1^	*K_a_* ^2^/10^−3^ s^−1^	References
NiPt-0.6% (Ni: 36.98 wt % Pt: 0.347 wt %)	15.5 mg, 2 mM, 25 °C, 0.25 M	18.82	This work
Pd_0.05_/G	4 mg, 0.3 mM, 25 °C, 0.1 M	36.5	[1]
Ir/IrO*_x_*	-, 20 mM, 25 °C, 0.2 M	2.57	[2]
230 nm Ni/SiO_2_ MHMs (Ni: 14.6 wt %)	3 mg, 5 mM, 25 °C, 0.2 M	4.5	[4]
Ni NPs	3 mg, 0.1mM, 20 °C, 0.2 M	2.7	[6]
Pd-Fe_3_O_4_ (1.2 wt %)	10 mg, 21.56 mM, 25 °C, 0.1 MPa	-	[7]
DPNs	-, 2 mM, 25 °C, 0.3 M	0.75	[11]
Ni/TiO_2_	400 mg, -, 100 °C, 1.5 MPa	-	[14]
Ni/SiO_2_@Au MHMs	4 mg, 5 mM, 25 °C, 0.2 M	10	[15]
RGO/PtNi (25:75)	3 mg, 5 mM, 25 °C, 1.5 M	1.12	[35]
TAC-Ag-1.0	0.004 mg, 0.103 mM, 25 °C, 0.3 M	5.19	[38]
Au-Cu alloy NP	50 mg, -, 60 °C, -	-	[39]
Pt/γ-Al_2_O_3_ (2.7 wt %)	0.5 mg, 1 mM, 22 °C, 0.1M	0.53	[40]
Ni-Pt (96:4)	0.004 mg, 0.085 mM, 25 °C, 0.012 M	1.93	[42]
AuNPs/SNTs	8 mg, 0.12 mM, 25 °C, 0.005 M	10.64	[43]
AuNP/CeO_2_ (Au: 0.031 mg)	10 mg, 0.12 mM, 25 °C, 0.005 M	2.25	[44]

^1^ Reaction conditions follow the order of amount of catalyst, the initial concentration of 4-NP, temperature, H_2_ pressure/the concentration of NaBH_4_; ^2^ *K_a_*: Apparent rate constant.
